# Supplementary material for: Bioisosteric Replacement in the Search for Biologically Active Compounds: Design, Synthesis and Anti-Inflammatory Activity of Novel [1,2,4]triazino[2,3-c]quinazolines
Source: Pharmaceuticals (Basel). 2024 Oct 28;17(11):1437. doi: 10.3390/ph17111437 (PMC11597805; doi:10.3390/ph17111437)
Supplement: Supplementary file 1 [file pharmaceuticals-17-01437-s001.zip › pharmaceuticals-3272646-supplementary.pdf]

Bioisosteric replacement in the search for biologically active compounds: design, synthesis and anti-inflammatory activity of novel [1,2,4]triazino[2,3-c]quinazolines.

Oleksandr Grytsak[1*], Konstantin Schabelnyk[1], Hanna Severina[2], Victor Ryzhenko[3], Oleksii Voskoboinik[4], Igor Belenichev[5], Serhiy Kovalenko[6], Valentyn Oksenych[7*], Oleksandr Kamyshnyi[8]

[1] Department of Pharmaceutical, organic and bioorganic chemistry, Zaporizhzhia State Medical and Pharmaceutical University, Zaporizhzhia, Ukraine;
[2] Department of Pharmaceutical chemistry, National University of Pharmacy, Kharkiv, Ukraine
[3] Department of medical and pharmaceutical informatics and modern technologies, Zaporizhzhia State Medical and Pharmaceutical University, Zaporizhzhia, Ukraine
[4] Department of Composite materials, chemistry and technologies, National University «Zaporizhzhia Polytechnic», Zaporizhzhia, Ukraine
[5] Department of Pharmacology and Medical Formulation with Course of Normal Physiology, Zaporizhzhia State Medical and Pharmaceutical University, 69000 Zaporizhzhia, Ukraine;

[6] Institute of Chemistry and geology, Oles Honchar Dnipro National University, Dnipro, Ukraine
[7] Department of Clinical Science, University of Bergen, 5020 Bergen, Norway
[8] Department of Microbiology, Virology and Immunology, I. Horbachevsky Ternopil State Medical University, 46001 Ternopil, Ukraine

* Correspondence: 2212dek2015@gmail.com (O.G.), valentyn.oksenych@uib.no (V.O.)

Content

^{1}H NMR-spectra of compound **2a**	**S3**
LC-MS spectra of compound **2b**	**S4**
^{1}H NMR-spectra of compound **2b**	**S5**
LC-MS spectra of compound **2c**	**S6**
^{1}H NMR-spectra of compound **2c**	**S7**
LC-MS spectra of compound **2d**	**S8**
^{1}H NMR-spectra of compound **2d**	**S9**
^{1}H NMR-spectra of compound **2e**	**S10**
LC-MS spectra of compound **2f**	**S11**

^{1}H NMR-spectra of compound **2f** **S12**

^{1}H NMR-spectra of compound **2g** **S13**

^{1}H NMR-spectra of compound **2h** **S14**

LC-MS spectra of compound **2i** **S15**

^{1}H NMR-spectra of compound **2i** **S16**

LC-MS spectra of compound **3a** **S17**

^{1}H NMR-spectra of compound **3a** **S18**

LC-MS spectra of compound **3b** **S19**

^{1}H NMR-spectra of compound **3b** **S20**

Table S1 **S21**

^{1}H NMR-spectra of compound **2a**

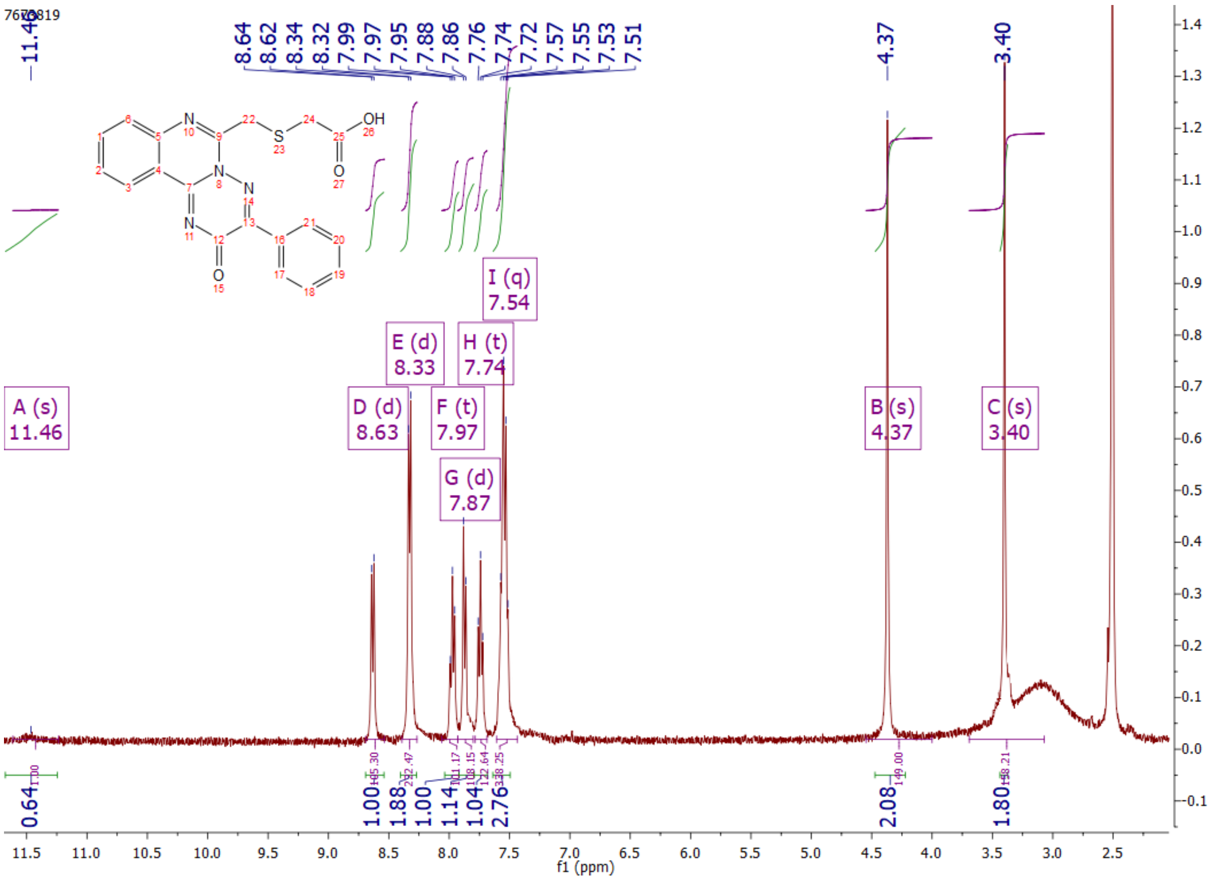

LC-MS spectra of compound **2b**

MaxPeak: 95.23%
Ret_Time: 1.190 min

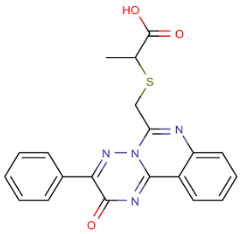

Mol Wt 392.43
Exact Mass 392.1

#	Time	Area%
1	1.042	4.77
2	1.190	95.23

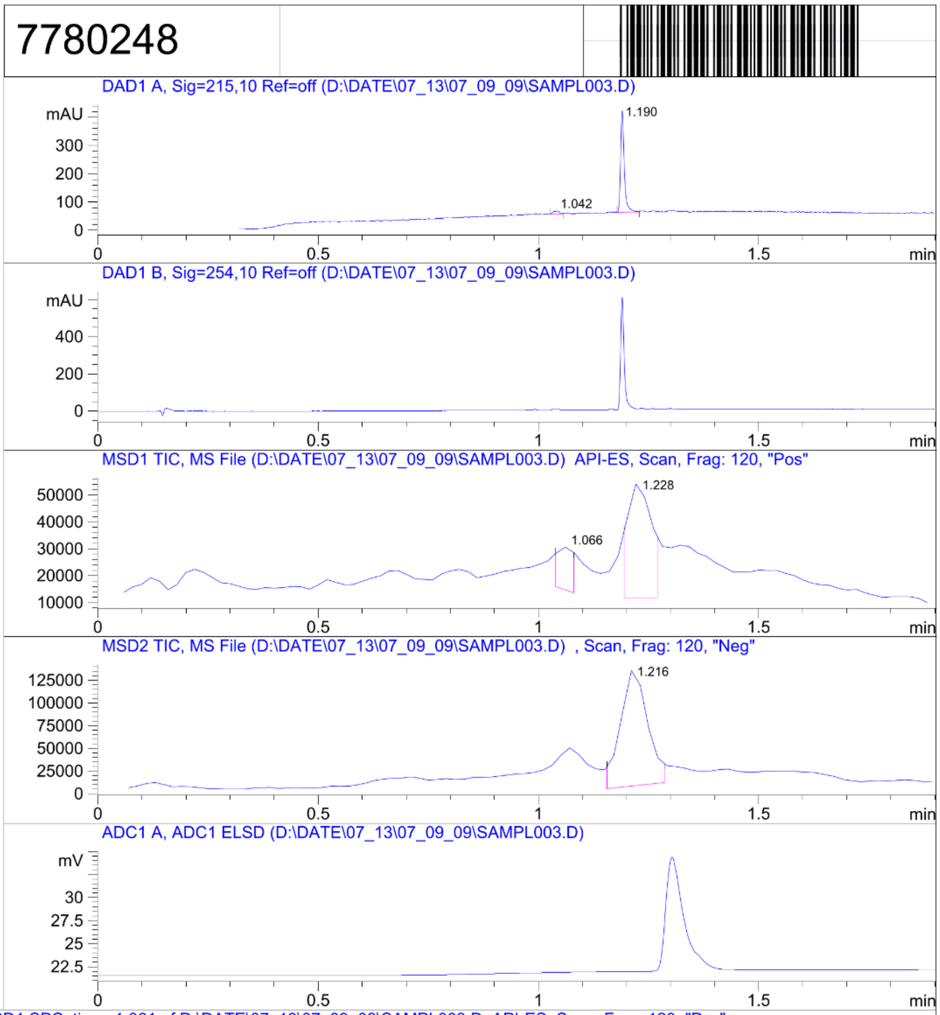

RT 1.066

RT 1.228

RT 1.216

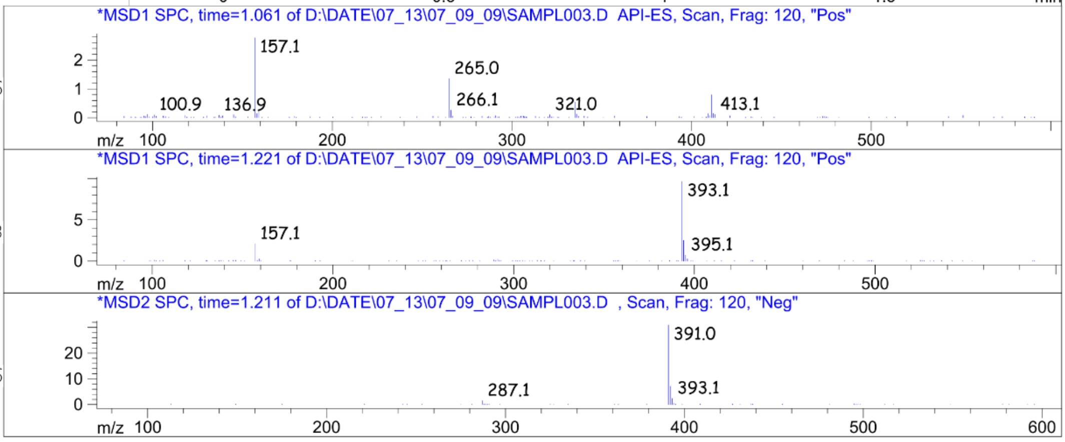

Inj.Date 7/12/2015 O P2-A-03 VL Acq. Method C:\HPCHEM\-> ->

^{1}H NMR-spectra of compound **2b**

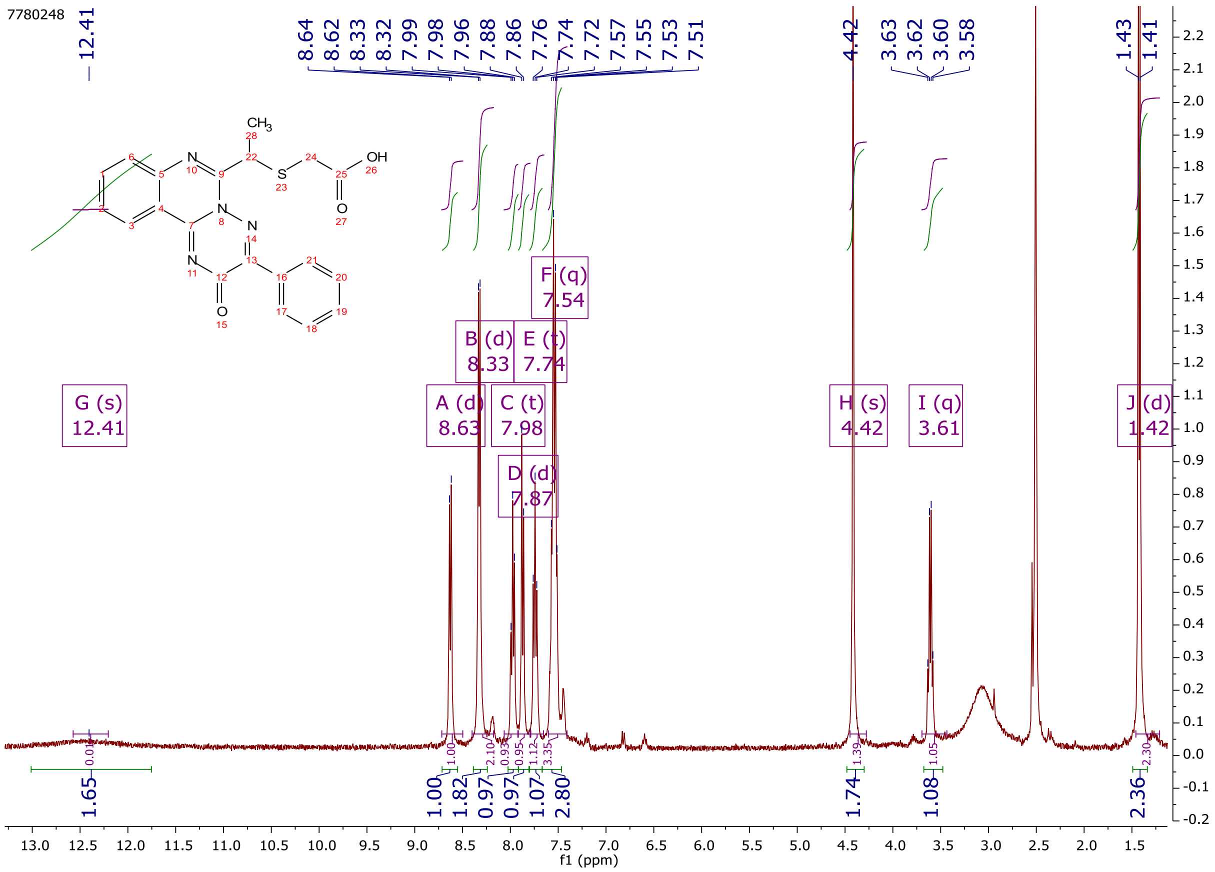

LC-MS spectra of compound **2c**

MaxPeak: 95.48%
Ret_Time: 1.467 min

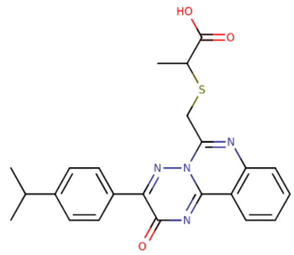

Mol Wt 434.51
Exact Mass 434.16

#	Time	Area%
1	1.467	95.48
2	1.549	3.39
3	1.809	1.13

RT 1.483

RT 1.482

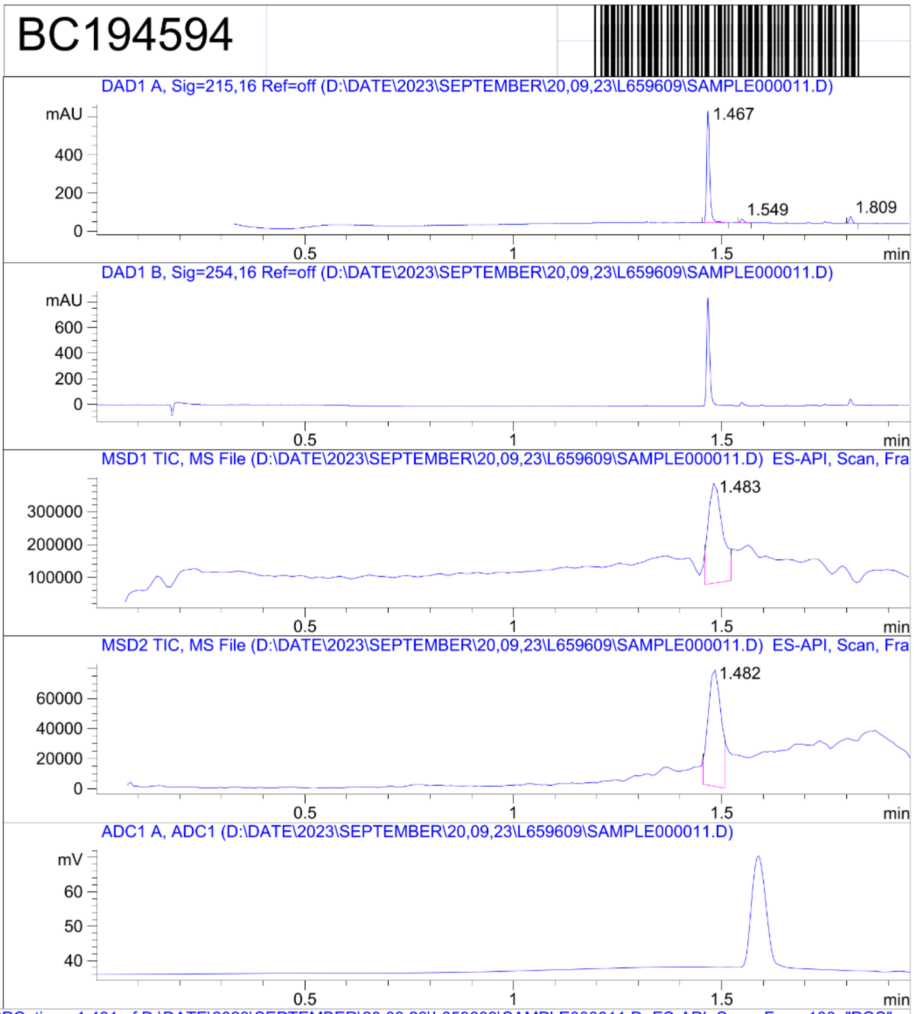

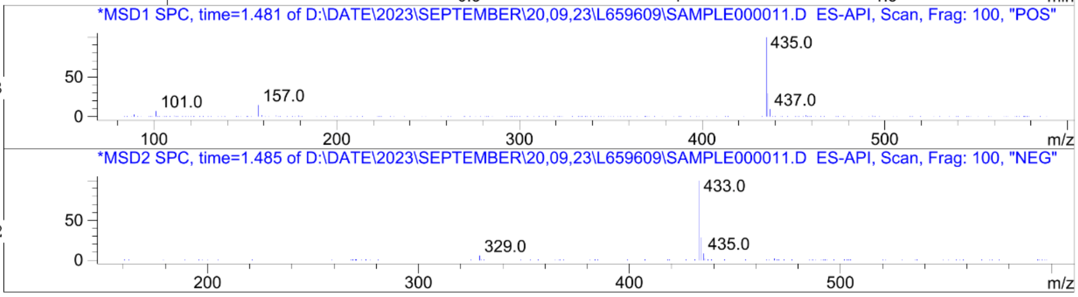

Inj.Date 9/20/2023 T P2-B-06 - 4 - Acq. Method C:\CHEM32\-> ->

^{1}H NMR-spectra of compound **2c**

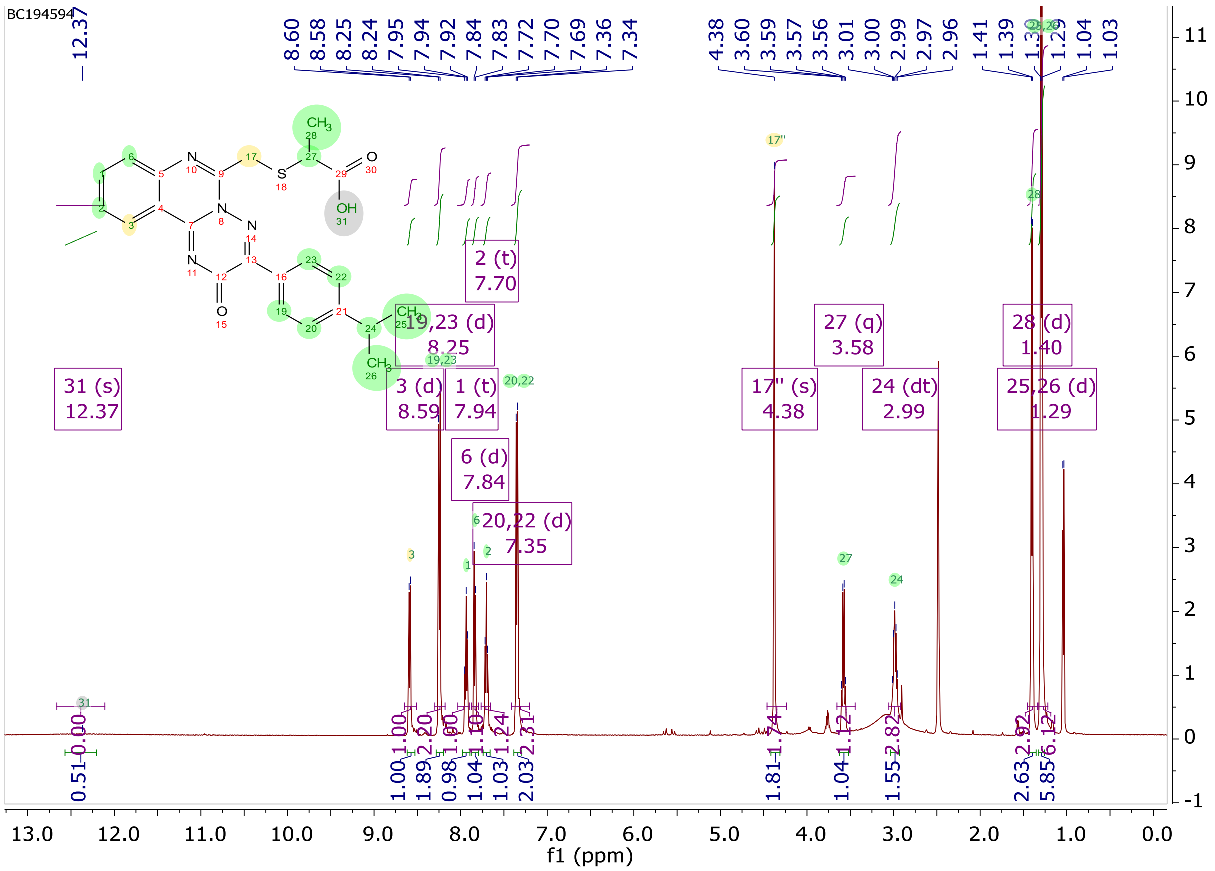

LC-MS spectra of compound **2d**

MaxPeak: 100.00%
Ret_Time: 1.236 min

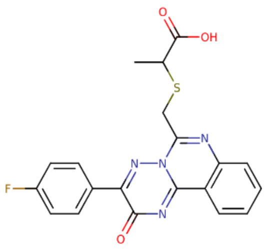

Mol Wt 410.42
Exact Mass 410.09

#	Time	Area%
1	1.236	100.00

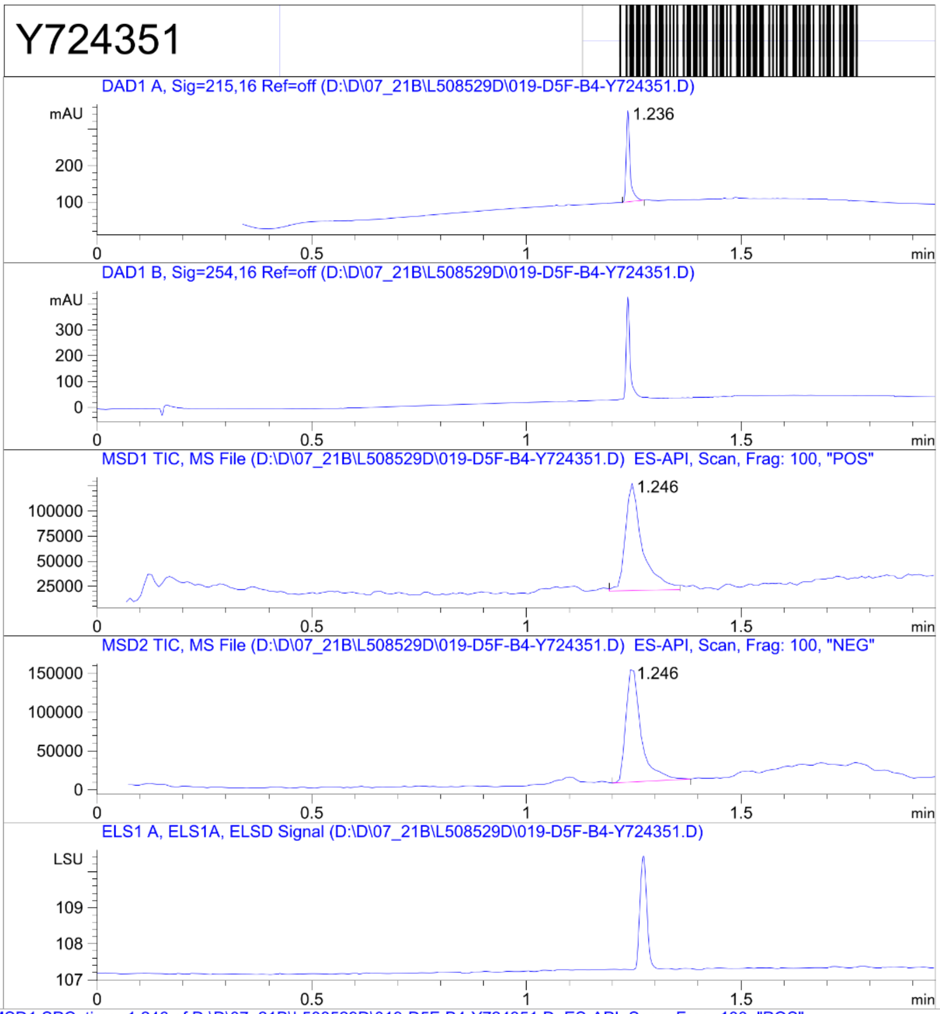

RT 1.246

RT 1.246

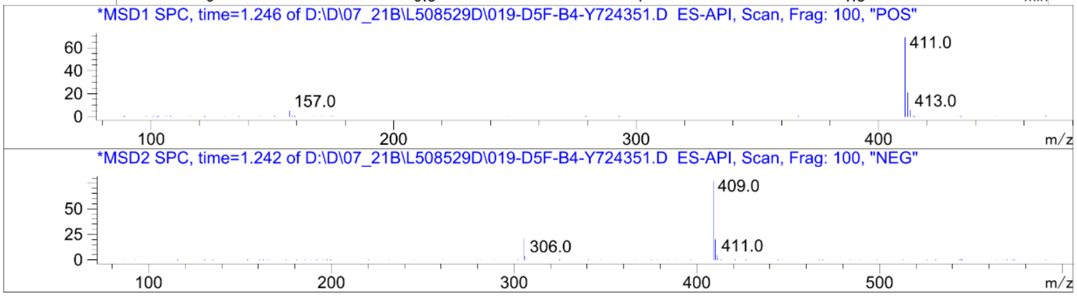

Inj.Date 7/21/2022 N -17- Acq. Method C:\Chem32\-> ->

^{1}H NMR-spectra of compound **2d**

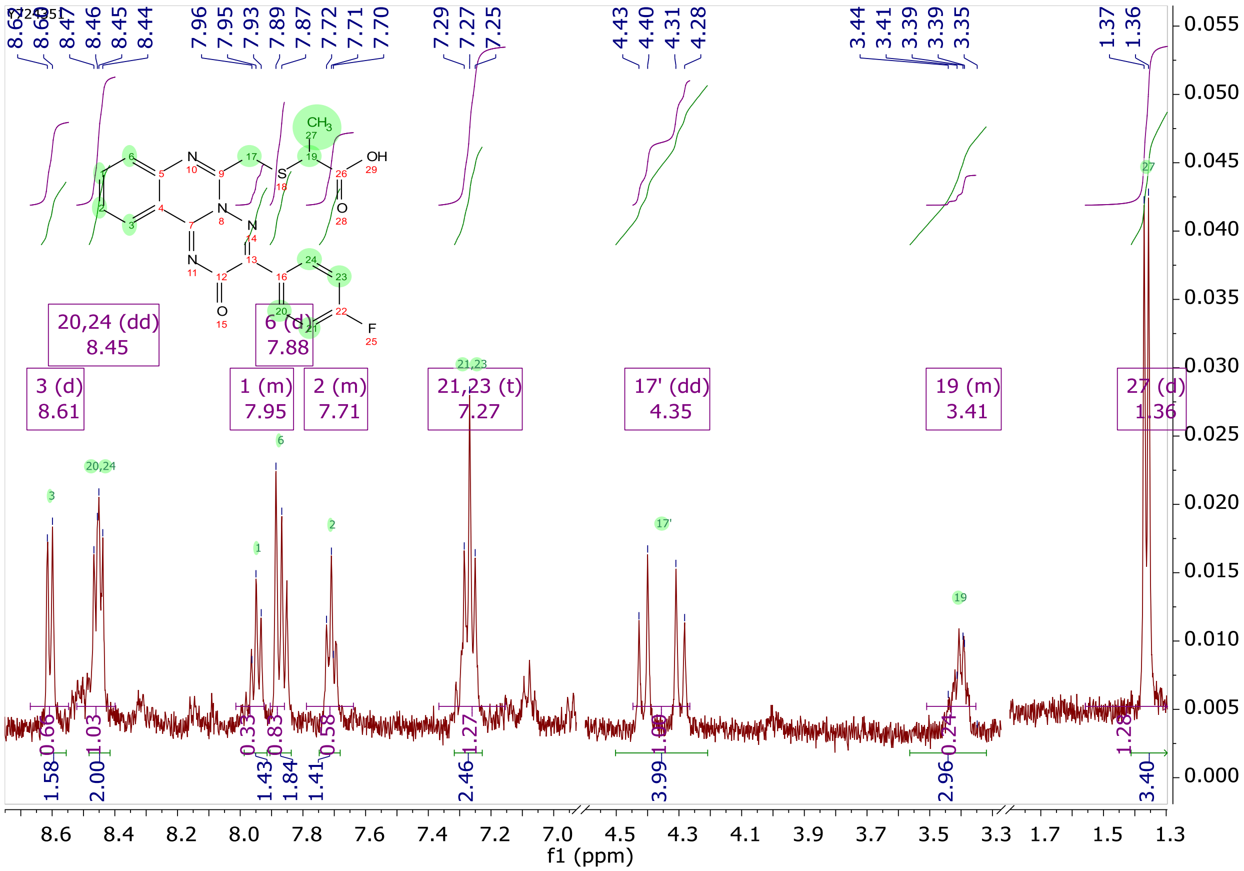

^{1}H NMR-spectra of compound **2e**

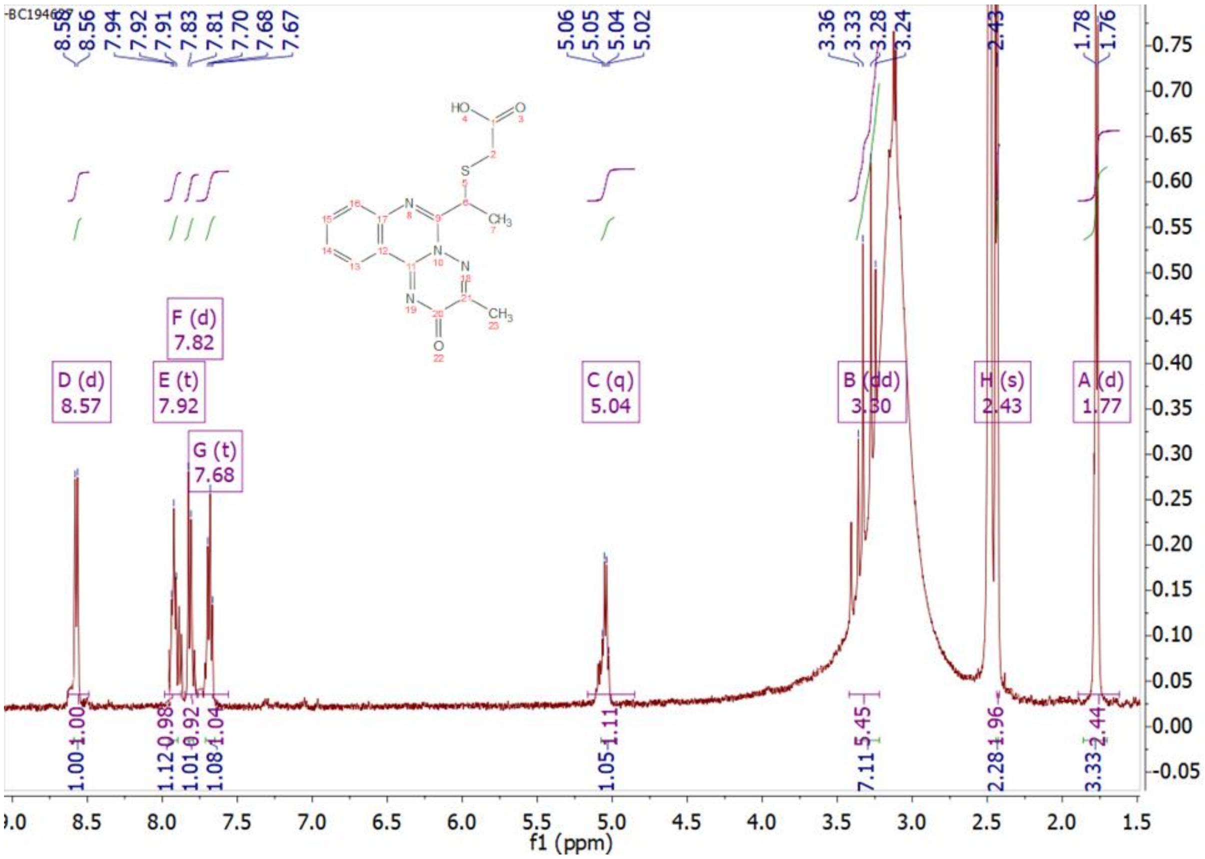

LC-MS spectra of compound **2f**

MaxPeak: 100.00%
Ret_Time: 1.220 min

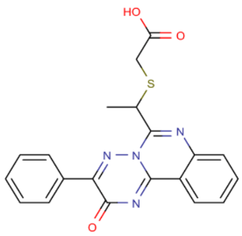

Mol Wt 392.43
Exact Mass 392.1

#	Time	Area%
1	1.220	100.00

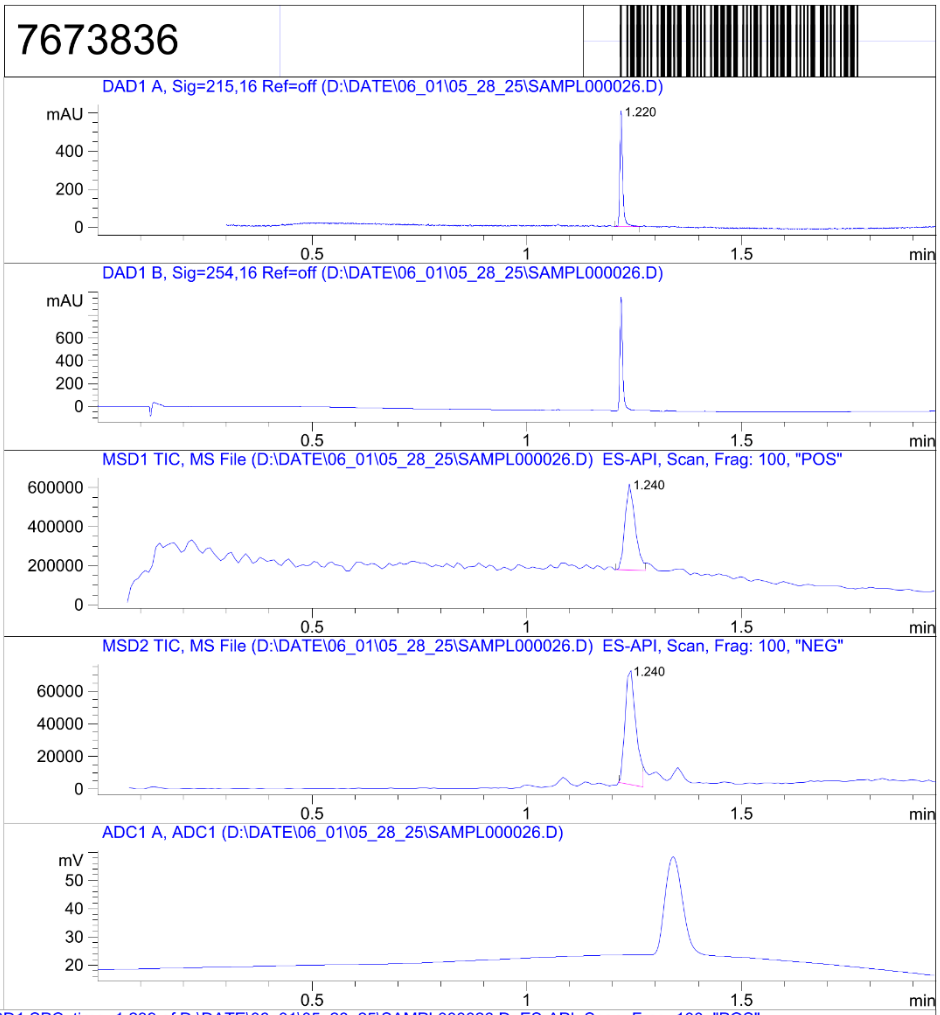

RT 1.240

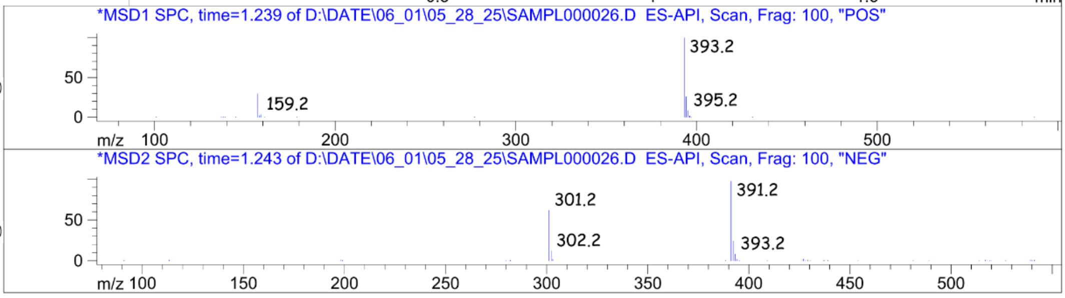

RT 1.240

Inj.Date 5/31/2015 O P2-D-02 '4' Acq. Method C:\CHEM32\-> ->

^{1}H NMR-spectra of compound **2f**

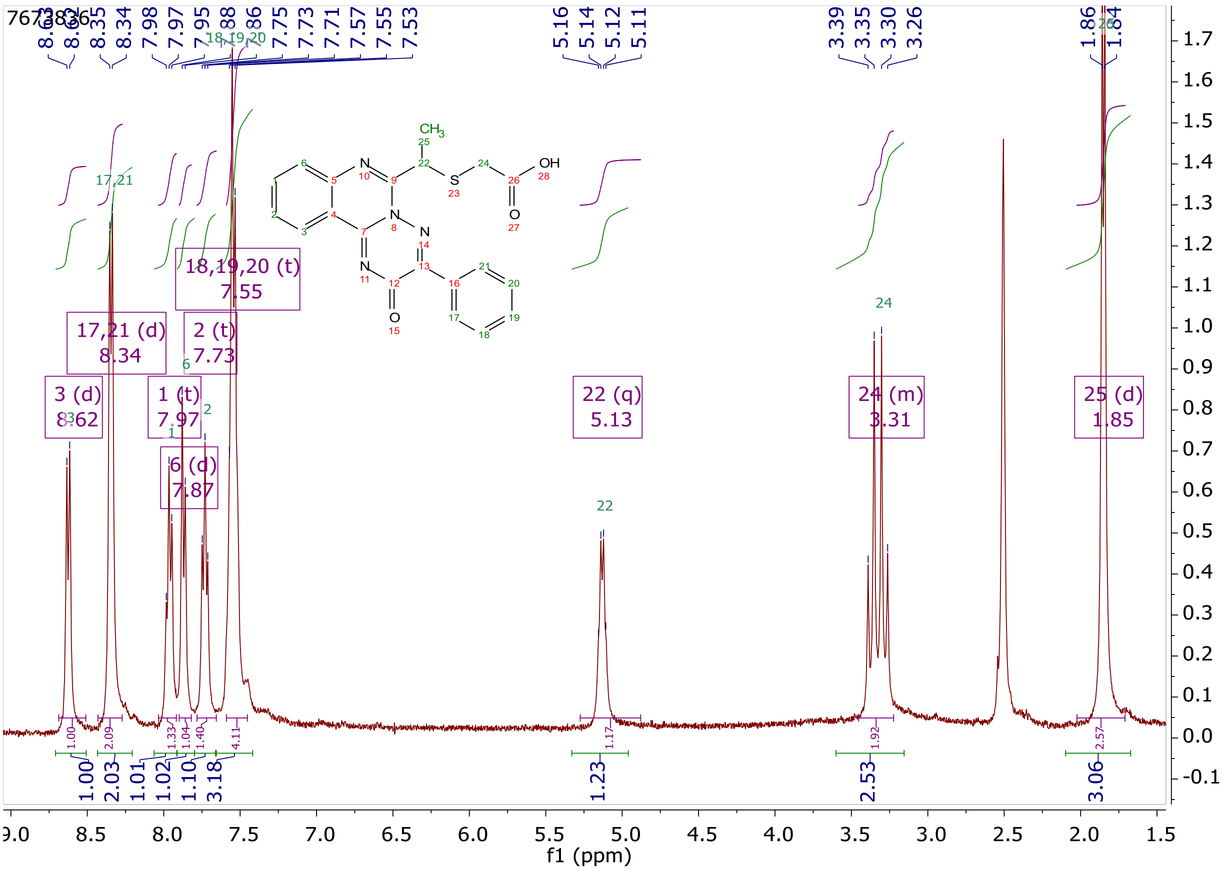

^{1}H NMR-spectra of compound **2g**

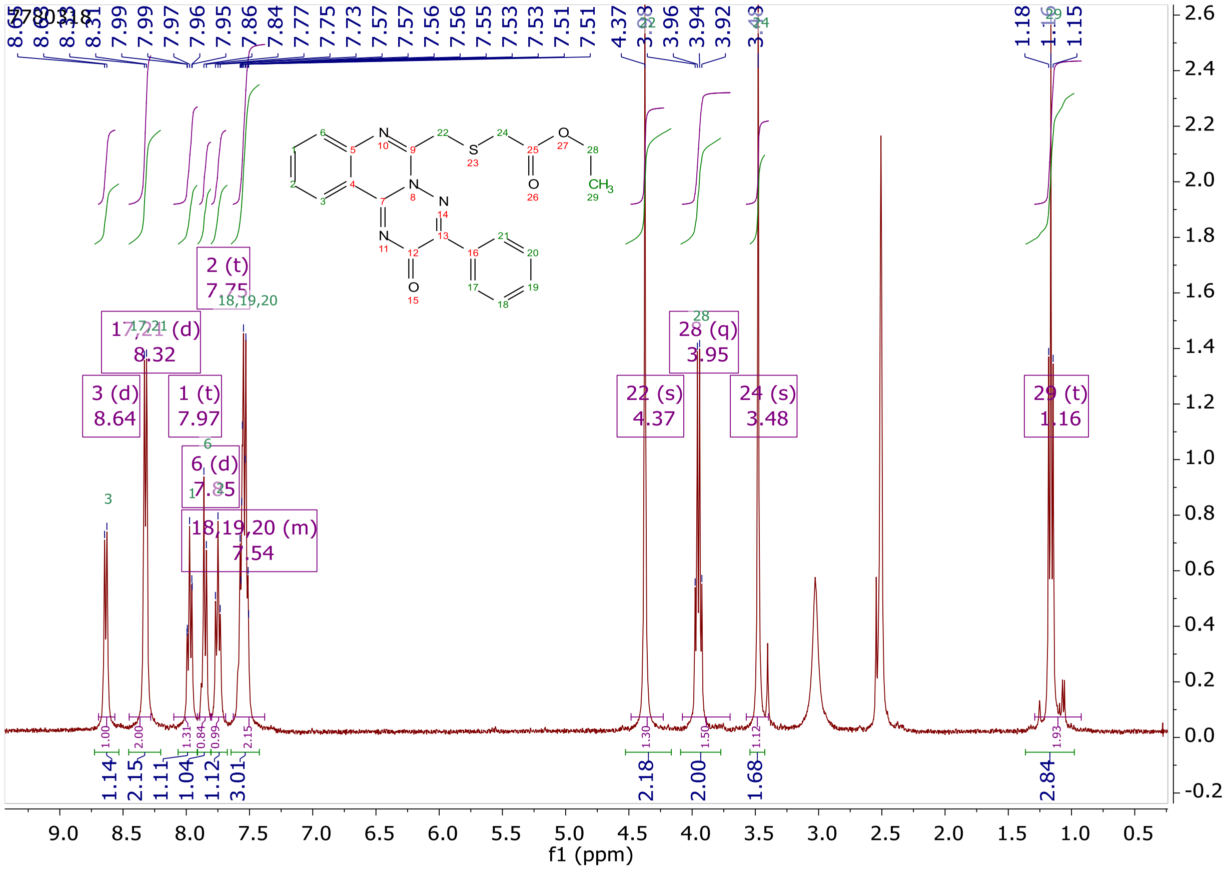

^{1}H NMR-spectra of compound **2h**

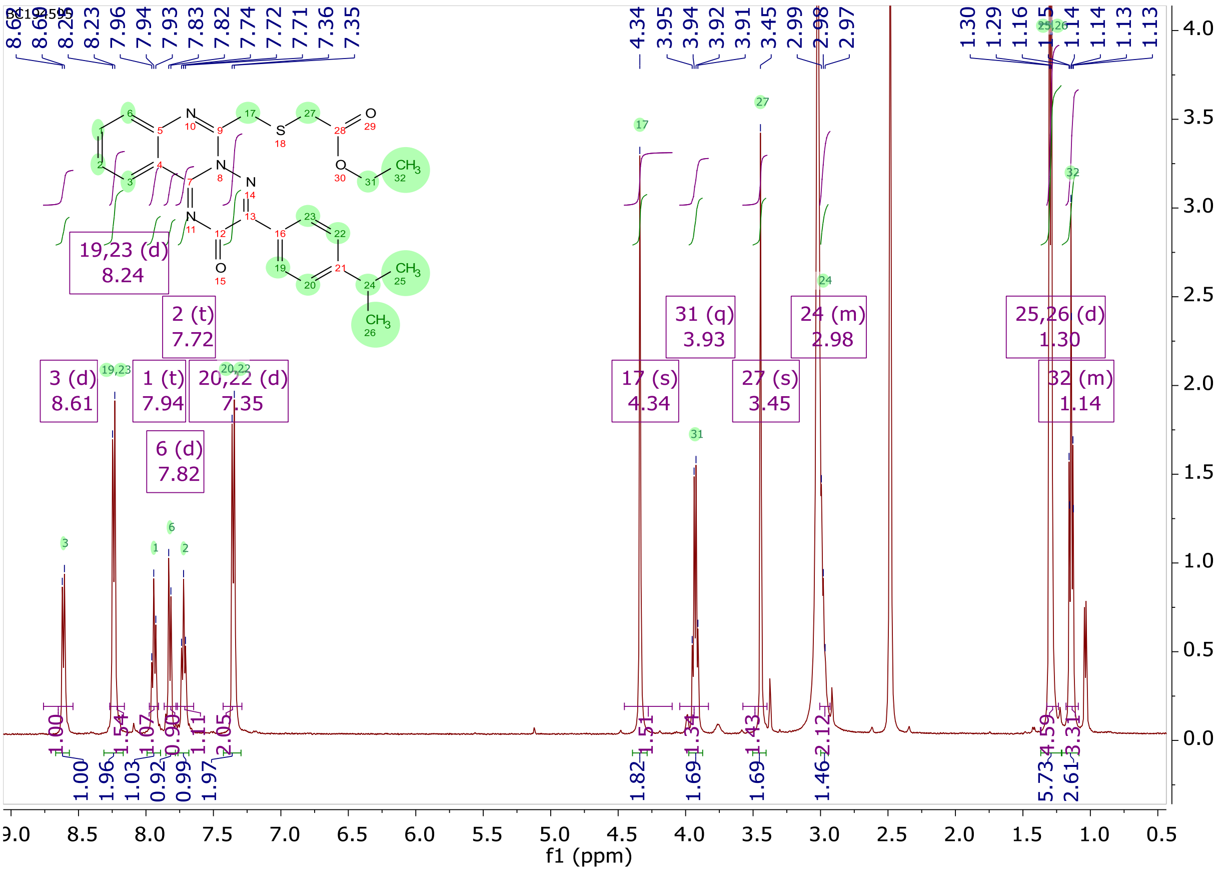

LC-MS spectra of compound **2i**

MaxPeak: 98.84%
Ret_Time: 1.388 min

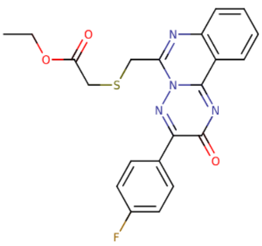

Mol Wt 424.45
Exact Mass 424.11

#	Time	Area%
1	1.167	1.16
2	1.388	98.84

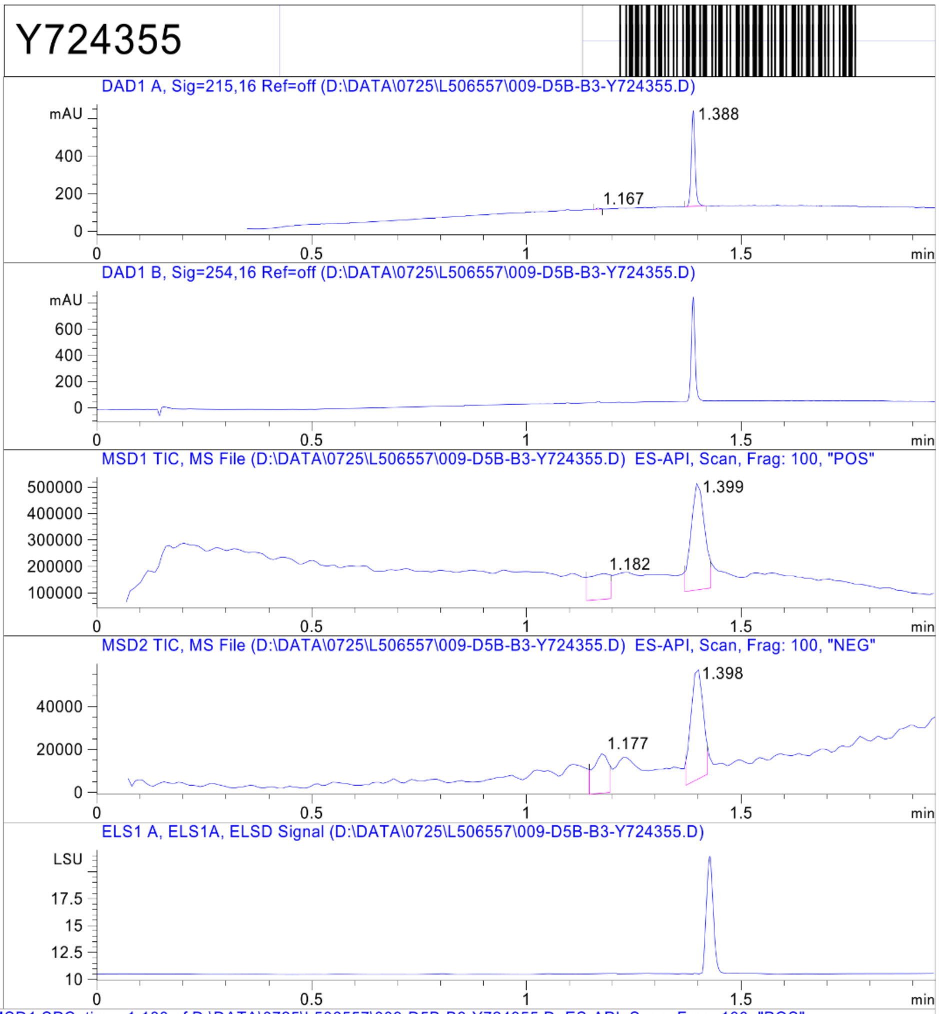

RT 1.182

RT 1.399

RT 1.177

RT 1.398

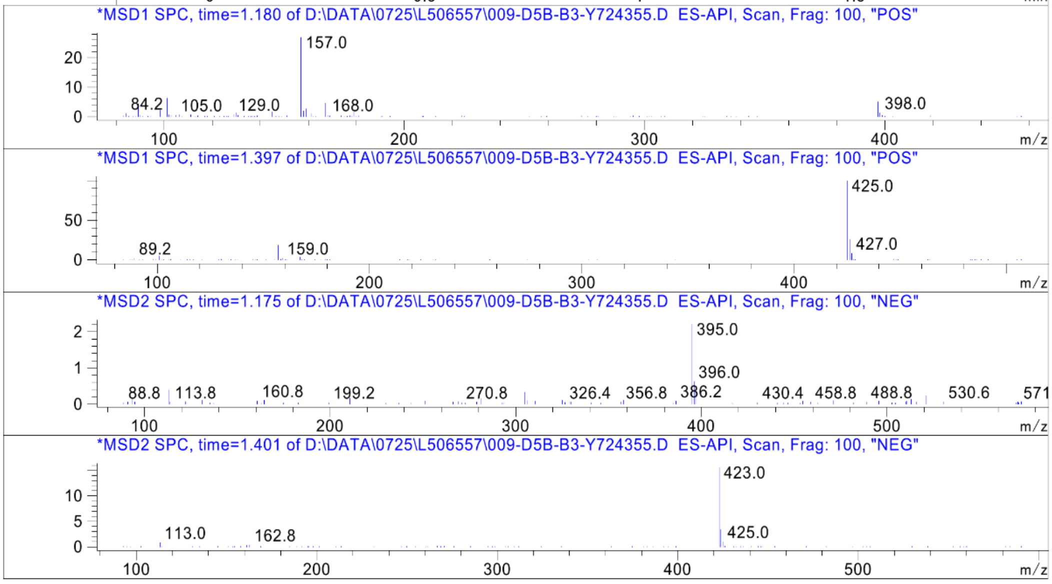

Inj.Date 7/17/2022 NK <invalid> 13 Acq. Method C:\Chem32\-> ->

^{1}H NMR-spectra of compound **2i**

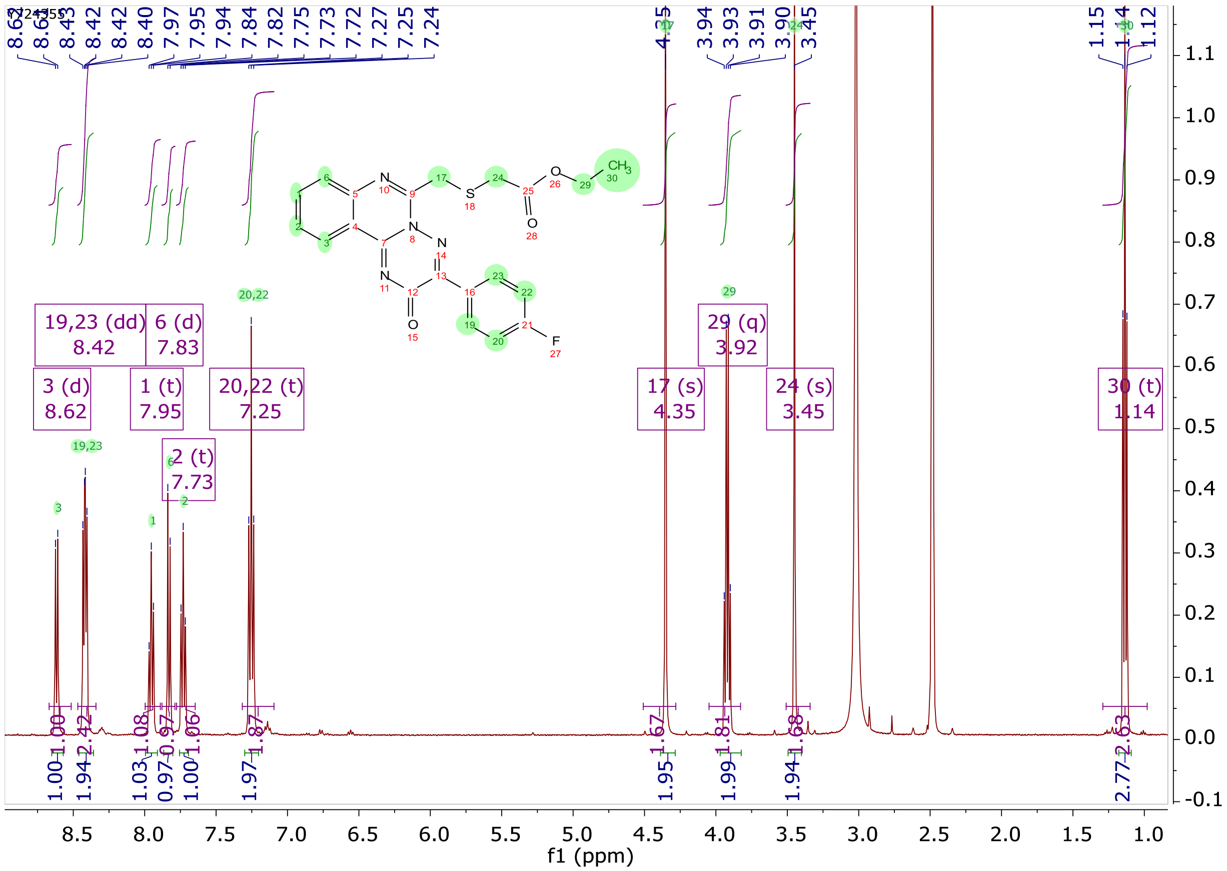

LC-MS spectra of compound **3a**

MaxPeak: 98.56%
Ret_Time: 1.067 min

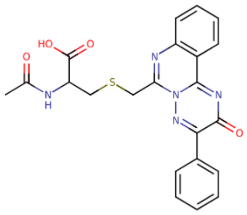

Mol Wt 449,49

Exact Mass 449,12

#	Time	Area%
1	0.969	1.44
2	1.067	98.56

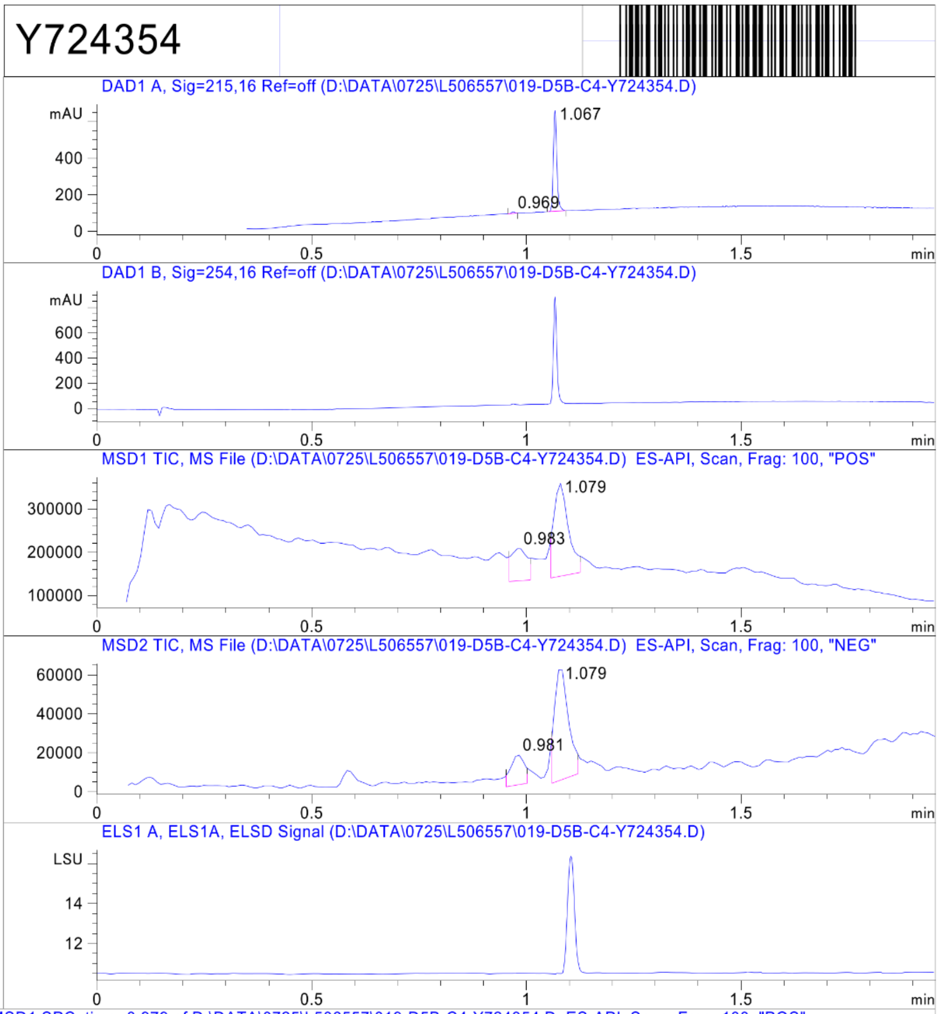

RT 0.983

RT 1.079

RT 0.981

RT 1.079

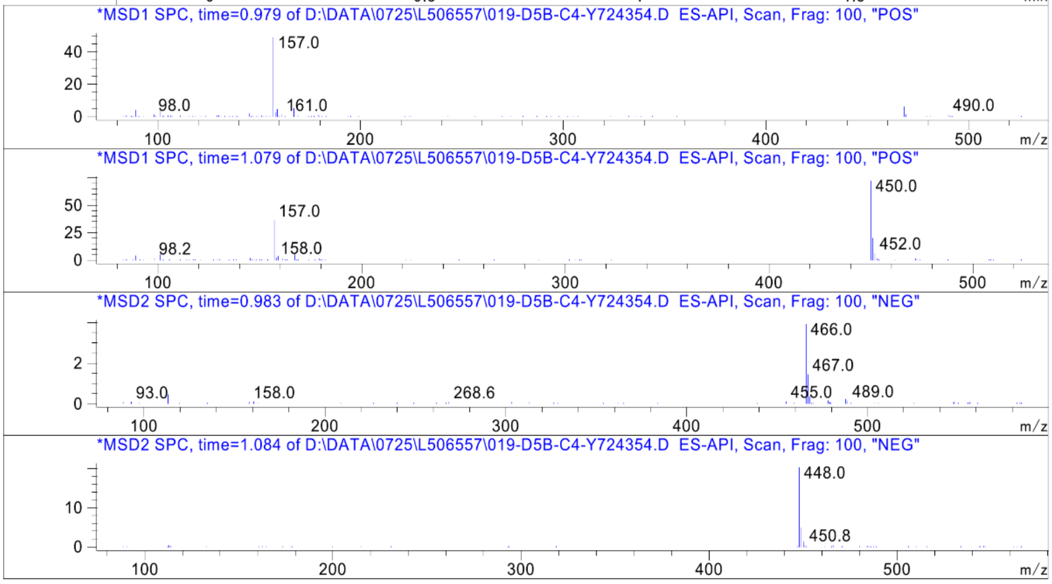

Inj.Date 7/17/2022   NK   <invalid> 13   Acq. Method C:\Chem32\-> ->

^{1}H NMR-spectra of compound **3a**

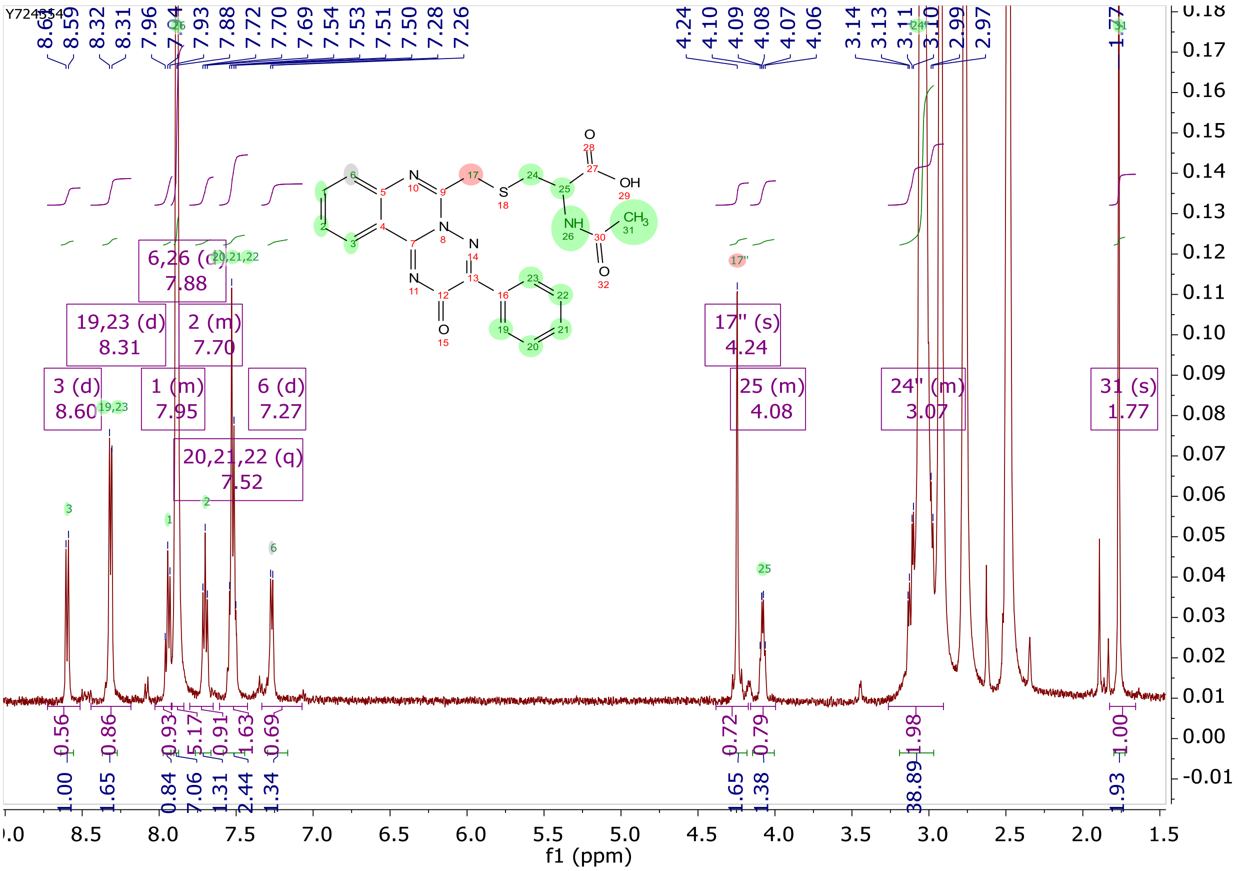

LC-MS spectra of compound **3b**

```
MaxPeak:  96.00%
Ret_Time: 1.342 min
```

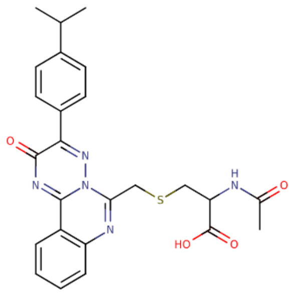

Mol Wt 491.56

Exact Mass 491.18

#	Time	Area%
1	1.263	4.00
2	1.342	96.00

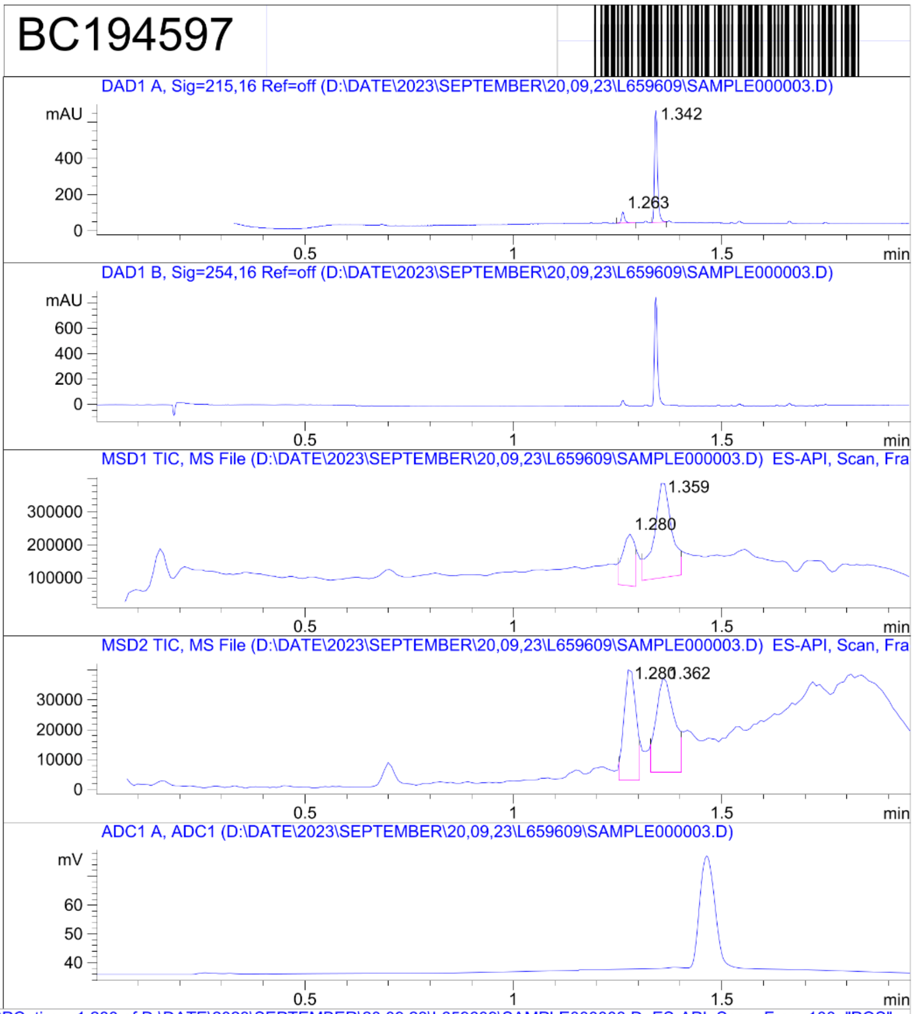

RT 1.280

RT 1.359

RT 1.280

RT 1.362

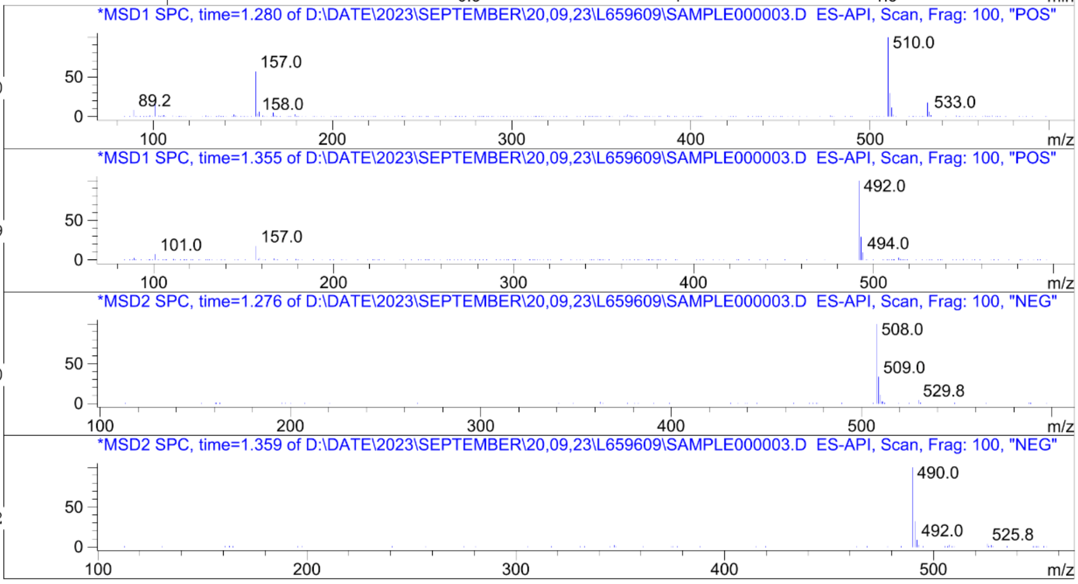


```
Inj.Date 9/20/2023          T       P2-A-02    - 4 -    Acq. Method C:\CHEM32\->    ->
```

^{1}H NMR-spectra of compound **3b**

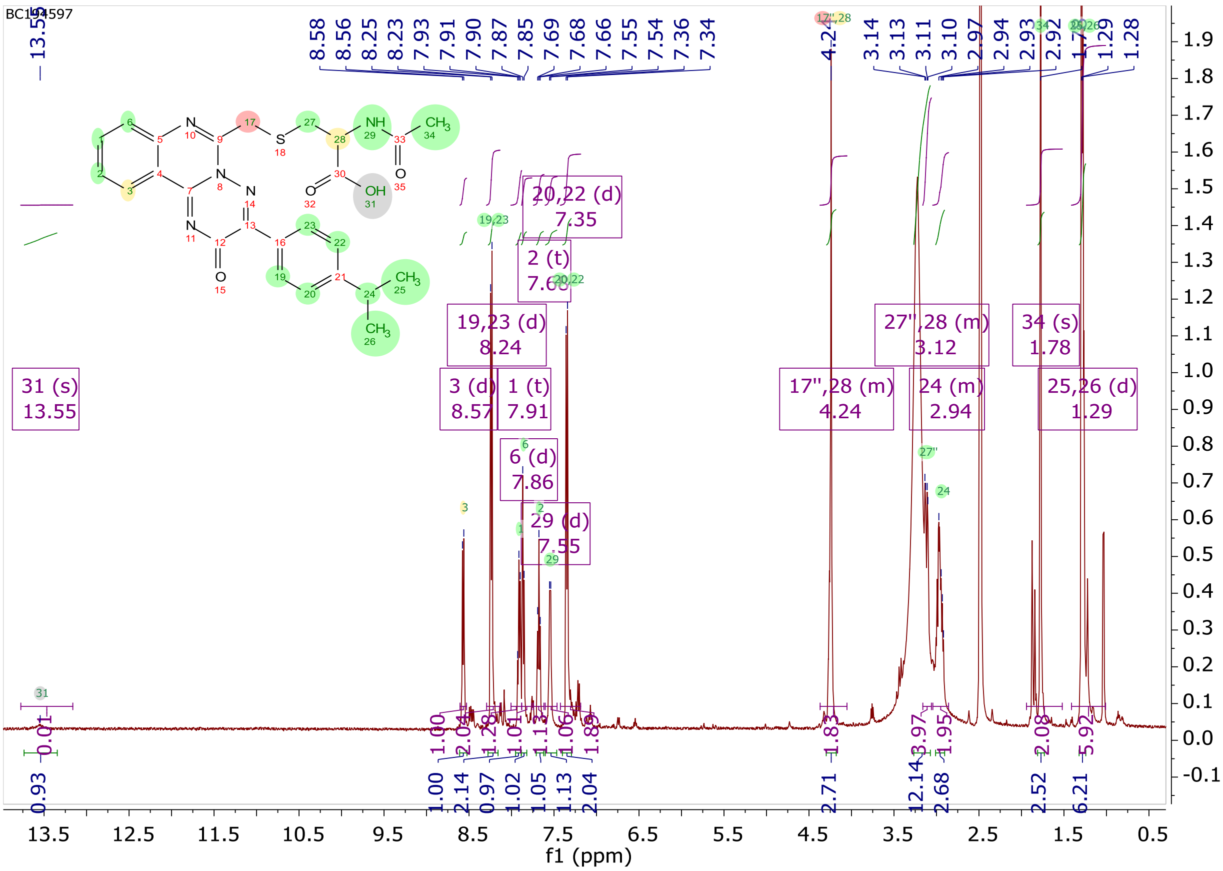

Table S1. The results of the docking studies of the ligand and the native inhibitors to the active site of COX-1 and COX-2.

Compounds	COX-1 amino acid moiety and type of the bonding with ligand	COX-2 amino acid moiety and type of the bonding with ligand
Diclofenac	a: Tyr385, Ser530<br>b: Ala527(3), Leu352(3), Ile523(2), Val349(2), Leu531	-
Celecoxib	-	a: Arg106, Arg499, Gln178, Leu338, Ser339<br>b: Val335, Ser339, Val509(2), Leu370, Val335, Leu345, Leu517, Tyr371, Trp373, Ala513(2)
MTB#	a: Ser530, Tyr385, Met522<br>b: Val349(3), Ala527(3), Leu352(2), Tyr348*, Tyr385, Trp387*, Leu531, Ile523	a: Tyr341(2), Ser516,<br>b: Val335, Tyr371, Val509(3), Ala513(2), Phe504, Val335
**2a**	a: Tyr355*, Tyr385, VAL349;<br>b: Leu352, Ala527(4), Met522*, Tyr348*, Gly526(3)*, Val349, Leu352, Ile523(2);<br>C: met113	a: Arg499, Ser516*, Ser339<br>b: Val509(2), Val335(3), Ala513(5), Leu517, Gly512(2)*, Val102*, Leu345<br>c: Met508*, Phe504*
**2b**	a: Tyr355*, Val116*<br>b: Val349, Leu352(2), Ala527(6), Met113*, Met522*, Tyr348*, Gly526(3)*, Val349(2), Ile523(2), Leu359*, Leu531	a: Ser516*, Gln178, Val509<br>b: Val335(2), Ala513 (6), Gly512 (3)*, Val509, Val102*, Leu345, Leu517<br>c: Met508*, Phe504*
**2c**	a: Arg120*, Tyr355*<br>b: Ile89(2*), Leu93*, Val116(3)*, Tyr355*, Ala527 (3), Val349(2), Leu531, Ile523	a: Phe504, Gln178<br>b: Tyr341(4)*, Val335(2), Leu338, Ala513(3), Leu78(2)*, Val102(3)*, Leu345(2), Phe343, Leu338, Val509
**2d**	a: Tyr355*<br>b: Val116*, Leu352(2), Ala527(5), Gly526(3)*, Val349(3), Ile523(2), Leu359*, Leu531<br>c: Met113*, Met522*, Tyr348*	a: Ser516*, Val509<br>b: Arg106, Ala513, Gly512(3)*, Ala513(5), Val509, Val335(4), Val102*, Leu345, Leu517<br>c: Met508*, Phe504*
**2e**	a: Arg120(2), Tyr355, Tyr385, Ser530;<br>b: Phe518*, Ala527(2), Val349(4), Leu531, Val344*, Leu534,* Tyr348*, Leu352<br>c: Met522*	a: Arg499, Ala513, Ser516*<br>b: Leu338, Val509(3), Trp373, Phe504 (2), Val335(2), Ala513(3), Leu517<br>c: Phe504, Met508
**2f**	a: Tyr355*, Tyr385<br>b: Leu352, Ala527(4), Met522*, Tyr348*, Gly526(3)*, Val349, Leu352, Ile523(2)	a: Tyr371, Ser516*, Val335, Val509, Ala513;<br>b: His75*, Met508, Val509(3), Phe504*, Val335, Ala513, Leu517, Arg499, Ala 502<br>c: Trp373 (π-Sulfur)
**2g**	a: Tyr355*, Tyr385(2)<br>b: Val116*, Val349(3), Leu352(2), Ala527(4), Met113*, Met522*, Tyr348*, Tyr385, Gly526(2)*, Ile523(2), Leu359*, Leu531	a: Arg499, Ser516*, His75*<br>b: Val335, Gly512(3)*, Ala513 (5), Val509, Val335, Val102*, Leu345, Leu517<br>c: Phe504*, Met508*
**2h**	a: Tyr385(2)	a: Arg499, His75*;

	b: Ala527(4), Phe518*, Tyr385*, Gly526*, Met113*, Val116(2)*, Leu359(2)*, Tyr355*, Leu352, Val349(2), Ile523, Leu384, Leu531<br>c: Met522*, Phe205*, Tyr348*	b: Ala513(6), Gly512(3)*, Leu78*, Val102(2), Leu345 (2), Tyr341(2)*, Phe343, Val509, Val335(3), Val102*, Leu517<br>c: Phe504*, Met508*
**2i**	a: Tyr385(2)<br>b: Ala527(6), Phe518*, Gly526 (3)*, Val349(2), Leu352(2), Ile523, Val116*, Leu359*, Leu531<br>c: Met522*, Tyr348*	a: Arg499, Ser516*<br>b: Arg106, Ala513, Gly512(3), Ala513(5), Val509, Val335(2), Val102*, Leu345, Leu517<br>c: Met508*, Phe504*
**3a**	a: Tyr355*;<br>b: Val349, Leu352(2), Ala527(6), Met113*, Met522*, Tyr348*, Gly526(3)*, Val349(2), Ile523(2)	a: Ser516*, Val509<br>b: Arg106, Ala513(6); Gly512(3)*, Val509, Val335(2), Val102*, Leu345, Leu517<br>c: Met508*, Phe504*
**3b**	a: Arg120*<br>b: Ser530, Val349(3), Leu531(3), Val116(2), Ile345, Leu359(2)*, Ala527(2)<br>c: Met113*, Tyr355*	a: Ser516*, Gln178<br>b: Arg106, Ala513, Gly512(3)*, Ala513(5), Val509, Val335(3), Val102*, Val335, Leu345, Leu517<br>c: Met508*, Phe504*

# – MTB - (3-methyl-2-oxo-2H-[1,2,4]triazino[2,3-c]quinazolin-6-yl)butanoic acids; a - Hydrogen Bond; b - Hydrophobic; c – other; () – the number of bonds is given in parentheses, * The amino acids which do not interact with native ligand in the experiment
